# Secondary analysis of malaria rapid diagnostic tests from rounds 5–8 of WHO product testing with a focus on false-negative results

**DOI:** 10.1186/s40779-021-00345-0

**Published:** 2021-10-07

**Authors:** Biao Xu, Bo Tu, Fang Chu, Mohamed Jalloh, Jin-Song Mu, Jun-Jie Zheng, Wei-Wei Chen

**Affiliations:** 1 Chinese Military Medical Expert Group in Sierra Leone, the Fifth Medical Center of Chinese PLA General Hospital, Beijing, 100039 China; 2 The 34Th Military Hospital, Wilberforce Barracks, Freetown, Sierra Leone

**Keywords:** Malaria, Rapid diagnostic tests, False-negative results, WHO product testing

## Abstract

Despite the widespread use of malaria rapid diagnostic test (RDT) in clinical practice, there are a lot of challenges. We conducted a secondary analysis of 129 malaria RDT data from rounds 5–8 of the World Health Organization (WHO) product testing summary and discuss the causes of false-negative (FN) results with a focus on low parasite density, improper RDT storage, operation and interpretation, and *plasmodium falciparum* with a *pfhrp2/3* gene deletion. The results demonstrated that the malaria RDTs currently commercially available might cause FN results in practice.

**Supplementary Information:**

The online version contains supplementary material available at 10.1186/s40779-021-00345-0.

Dear Editor,

Malaria rapid diagnostic test (RDT) is becoming the most-used method to diagnose malaria worldwide, especially in sub-Saharan Africa because malaria RDTs are easier to utilize than microscopy and have been shown to have comparable detection capability in the field [1]. Despite the widespread use of RDTs in the clinical setting, there are challenges, such as false-positive (FP) and false-negative (FN) results [2]. The World Health Organization (WHO) has conducted a systematic evaluation of the performance of commercially-available malaria RDTs since 2008, in which FP results were regarded as an important evaluation index, but FN results were not mentioned [2]. Due to the actual clinical needs, we conducted a secondary analysis of the 5th–8th rounds of the WHO malaria RDT product testing and discuss the causes of FN results.

There were 129 RDT products enrolled into the product assessment on malaria RDT performance in this summary [3]. The summarized data were analyzed based on four measures, including low parasite density, improper RDT storage, operation and interpretation, and *Plasmodium falciparum* (Pf) with a *pfhrp2/3* gene deletion.

Low-density malaria infection is common among populations in endemic settings and potentially contributes to ongoing malaria transmission. Plucinski et al. [4] analyzed 207 outpatient samples from Angola in 2019 and showed that among histidine-rich protein 2 (HRP2)-positive patients with negative RDT results, the positive rate of quantitative RT-PCR was 45% (95% CI 35–56%). As shown in this analysis, the proportion of tests that had a panel detection score (PDS) < 80% at a low parasite density was 20–25% for Pf, which is substantially higher for *Plasmodium vivax* (Pv; Fig. 1). The results indicated that the RDT products with a lower PDS are likely to produce FN results.

**Fig. 1:**
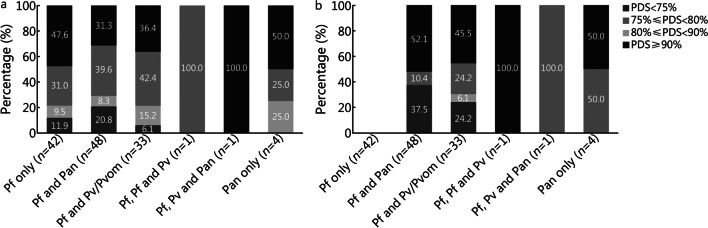
Assessment of malaria RDT performance in rounds 5–8 against wild type (clinical) samples containing Pf (**a**) and Pv (**b**) at 200 parasites/µl (%). Pf *Plasmodium falciparum*, Pv *Plasmodium vivax*, Pan all *Plasmodium* species, Pvom *Plasmodium* vivax, ovale and malariae

Malaria RDTs are based on capturing parasite antigen with antibodies stabilized on a nitrocellulose (organic) strip. The storage of RDTs for prolonged periods in hot or humid conditions may impair the diagnostic ability. This analysis showed that the positive rate of some products at low parasite density was impaired as the incubation temperature increased. Malaria is endemic in some geographic areas, such as sub-Saharan Africa and Southeast Asia, which are characterized by a hot, humid climate and poor storage infrastructures. As a result, RDTs were usually exposed to temperatures and humidity above the product recommended limits in these endemic areas, which may result in poor test performance.

RDTs are considered to be an ideal way for parasite-based diagnosis, particularly in remote and resource-limited areas, mainly due to the simplicity of the test. The results of this analysis showed that approximately 20–27% of products need to be improved for blood safety and instruction quality, 30–35% of RDT test bands against Pf were minimally visible, and that the RDT test bands against Pv returned a higher proportion (Additional file 1: Table S1 and Table S2). Seidahmed et al. [5] also reported that the specific errors caused by test design and manufacturer's instructions significantly impair the accuracy of the malaria RDTs in a meso-endemic area of eastern Sudan.

The parasites have no PfHRP2 protein because the *pfhrp2* gene deletion is not recognized by the RDT antigens against PfHRP2. Given the absence of PfHRP2 protein, most of the antibodies used in RDTs for the detection of PfHRP2 also detect the PfHRP3 protein due to structural homology. Some of the parasites have combined deletions of the *pfhrp2* and *pfhrp3* genes, which result in FN results. Moreover, the parasites with deletion of the *pfhrp2/3* gene have less competition within the host and an increased probability of transmission success. The analysis showed that for HRP2-negative Pf, most malaria RDTs have a PDS < 50% and a high FN rate (Additional file 1: Table S1). In addition, the WHO report [3] indicated that positive results on the Pf–detecting test bands were obtained against HRP2-negative samples and the band intensities were generally weak, which lead to a higher risk of FN for malaria RDTs.

In summary, the currently widely used malaria RDTs can yield FN results. Clinicians and epidemic prevention personnel must clearly understand the shortcomings of RDTs and accurately interpret those results to draw accurate judgments and treatments, and maximize the effectiveness of RDT products.

## Supplementary Information

**Additional file 1.** **Table S1.** Percentage distribution of test band intensity score against wild-type Pf in 200 parasites/μl (%). **Table S2.** Percentage distribution of test band intensity score against wild-type Pv in 200 parasites/μl (%).

## Data Availability

The datasets generated and analyzed in this study are available in Summary results of WHO product testing of malaria RDTs-Round published online of WHO homepage (https://www.who.int/malaria/publications/atoz/9789241514965/en/-68).

## Abbreviations

FP: False-positive
FN: False-negative
HRP-2: Histidine-rich protein-2
PDS: Panel detection score
Pf: *Plasmodium falciparum*
Pv: *Plasmodium vivax*
RDT: Rapid diagnostic test
WHO: World Health Organization
