# Supplementary material for: Secondary analysis of malaria rapid diagnostic tests from rounds 5–8 of WHO product testing with a focus on false-negative results
Source: Mil Med Res. 2021 Oct 7;8:52. doi: 10.1186/s40779-021-00345-0 (PMC8496075; doi:10.1186/s40779-021-00345-0)
Supplement: Supplementary file 1 — Additional file 1. Table S1. Percentage distribution of test band intensity score against wild-type Pf in 200 parasites/μl (%). Table S2. Percentage distribution of test band intensity score against wild-type Pv in 200 parasites/μl (%). [file 40779_2021_345_MOESM1_ESM.docx]

**Table S1** Percentage distribution of test band intensity score against wild-type Pf in 200 parasites/µl (%)

| Products category | Test band intensity score | | | | |
| --- | --- | --- | --- | --- | --- |
|  | 0^*^ | 1 | 2 | 3 | 4 |
| Pf test band |  |  |  |  |  |
| Pf only (*n* = 44^a^) | 9.6 ± 12.0 | 19.4 ± 11.5 | 32.6 ± 9.0 | 23.8 ± 9.2 | 14.7 ± 11.2 |
| Pf and Pan（*n* = 48） | 12.4 ± 10.9 | 22.6 ± 10.9 | 32.5 ± 10.1 | 22.7 ± 9.9 | 9.8 ± 9.0 |
| Pf and Pv/Pvom (*n* = 33) | 8.5 ± 4.8 | 19.6 ± 8.3 | 34.2 ± 6.0 | 26.7 ± 8.5 | 11.2 ± 7.7 |
| Pf, Pf and Pv (*n* = 3) | 16.8 ± 14.8 | 39.4 ± 33.4 | 22.3 ± 17.7 | 12.2 ± 17.2 | 9.5 ± 13.4 |
| Pf, Pv and Pan (*n* = 1) | 5.8 | 12.0 | 27.3 | 31.5 | 23.5 |
| Pan test band |  | | | | |
| Pf and Pan (*n* = 48) | 52.9 ± 27.0 | 40.6 ± 21.7 | 6.5 ± 7.6 | 0.1 ± 0.2 | 0.0 ± 0.0 |
| Pf, Pv and Pan (*n* = 1) | 14.0 | 53.3 | 32.0 | 0.8 | 0 |
| Pan only (*n* = 4) | 6.0 ± 7.3 | 42.2 ± 24.1 | 33.6 ± 9.9 | 14.2 ± 16.2 | 4.1 ± 4.7 |
| Pv test band |  | | | | |
| Pf and Pv/Pvom (*n* = 33) | 95.6 ± 10.5 | 4.2 ± 10.0 | 0.3 ± 0.8 | 0.0 ± 0.0 | 0.0 ± 0.1 |
| Pf, Pv and Pan (*n* = 1) | 99.3 | 0.8 | 0 | 0 | 0 |

^*^Denotes no visible band.

^a^In the 8th round of testing, two bands HRP2 and pLDH of RMSM-02571, 05FK90 were tested respectively in 42 products of PF only during the assessment of test band intensity, which resulted in 44 analysis results.

*Pf* *Plasmodium falciparum*, *Pv* *Plasmodium vivax*, *Pan* all *Plasmodium* species, *Pvom* Plasmodium vivax, ovale and malariae

**Table S2** Percentage distribution of test band intensity score against wild-type Pv in 200 parasites/µl (%)

| Products category | Test band intensity score | | | | |
| --- | --- | --- | --- | --- | --- |
|  | 0* | 1 | 2 | 3 | 4 |
| Pf test band |  |  |  |  |  |
| Pf only (*n* = 39^a^) | 99.4 ± 1.3 | 0.6 ± 1.3 | 0.0 ± 0.0 | 0.0 ± 0.1 | 0.0 ± 0.0 |
| Pf and Pan (*n* = 32) | 99.6 ± 0.9 | 0.3 ± 0.8 | 0.0 ± 0.2 | 0.1 ± 0.2 | 0.0 ± 0.0 |
| Pf and Pv/Pvom (*n* = 29) | 99.4 ± 1.4 | 0.6 ± 2.4 | 0.0 ± 0.1 | 0.0 ± 0.0 | 0.0 ± 0.0 |
| Pf, Pf and Pv (*n* = 2) | 100.0 ± 0.0 | 0.0 ± 0.0 | 0.0 ± 0.0 | 0.0 ± 0.0 | 0.0 ± 0.0 |
| Pf, Pv and Pan (*n* = 1) | 99.3 | 0.7 | 0 | 0 | 0 |
| Pan test band |  | | | | |
| Pf and Pan (*n* = 48) | 13.1 ± 21.0 | 39.4 ± 24.0 | 42.3 ± 28.1 | 5.0 ± 7.5 | 0.3 ± 0.7 |
| Pf, Pv and Pan (*n* = 1) | 0 | 18.6 | 70.7 | 10 | 0.7 |
| Pan only (*n* = 4) | 2.7 ± 3.2 | 11.4 ± 7.2 | 65.9 ± 5.5 | 17.9 ± 5.9 | 2.1 ± 1.6 |
| Pv test band |  | | | | |
| Pf and Pv/Pvom (*n* = 33) | 6.9 ± 8.0 | 45.1 ± 22.5 | 42.6 ± 20.7 | 5.2 ± 10.6 | 0.2 ± 0.4 |
| Pf, Pf and Pv (*n* = 1) | 0.7 | 23.6 | 75.7 | 0 | 0 |
| Pf, Pv and Pan (*n* = 1) | 9.3 | 49.3 | 39.3 | 2.1 | 0 |

^*^Denotes no visible band

^a^In the 5th round of testing, the band intensity evaluation for Pv testing did not include the 5 RDT products only for Pf (IR016025, I13FRC, 0502_K25, KH-R-06020, 05FK50/05FK53), which resulted in 39 results.

*Pf* *Plasmodium falciparum*, *Pv* *Plasmodium vivax*, *Pan* all *Plasmodium* species, *Pvom* Plasmodium vivax, ovale and malariae
